# Supplementary material for: A yeast phenomic model for the influence of Warburg metabolism on genetic buffering of doxorubicin
Source: Cancer Metab. 2019 Oct 23;7:9. doi: 10.1186/s40170-019-0201-3 (PMC6806529; doi:10.1186/s40170-019-0201-3)
Supplement: Supplementary file 9 — Additional file 9. GO term-specific heatmaps for REMc/GTF-enriched clusters. GO term-specific heatmaps for significant GO process terms were generated as described in methods and Figs. 3 and 4. Any related child terms are presented in subsequent pages of the parent file name. GO terms with more than 100 children, with 2 or fewer genes annotated to the term, or a file size over 300KB are not shown. All heatmaps are generated with the same layout (see Figs. 3 and 4). [file 40170_2019_201_MOESM9_ESM.bz2 › Additional_File9_GOTermHeatmaps/Additional_File9_GOTermHeatmap/supramolecular_fiber_organization.pdf]

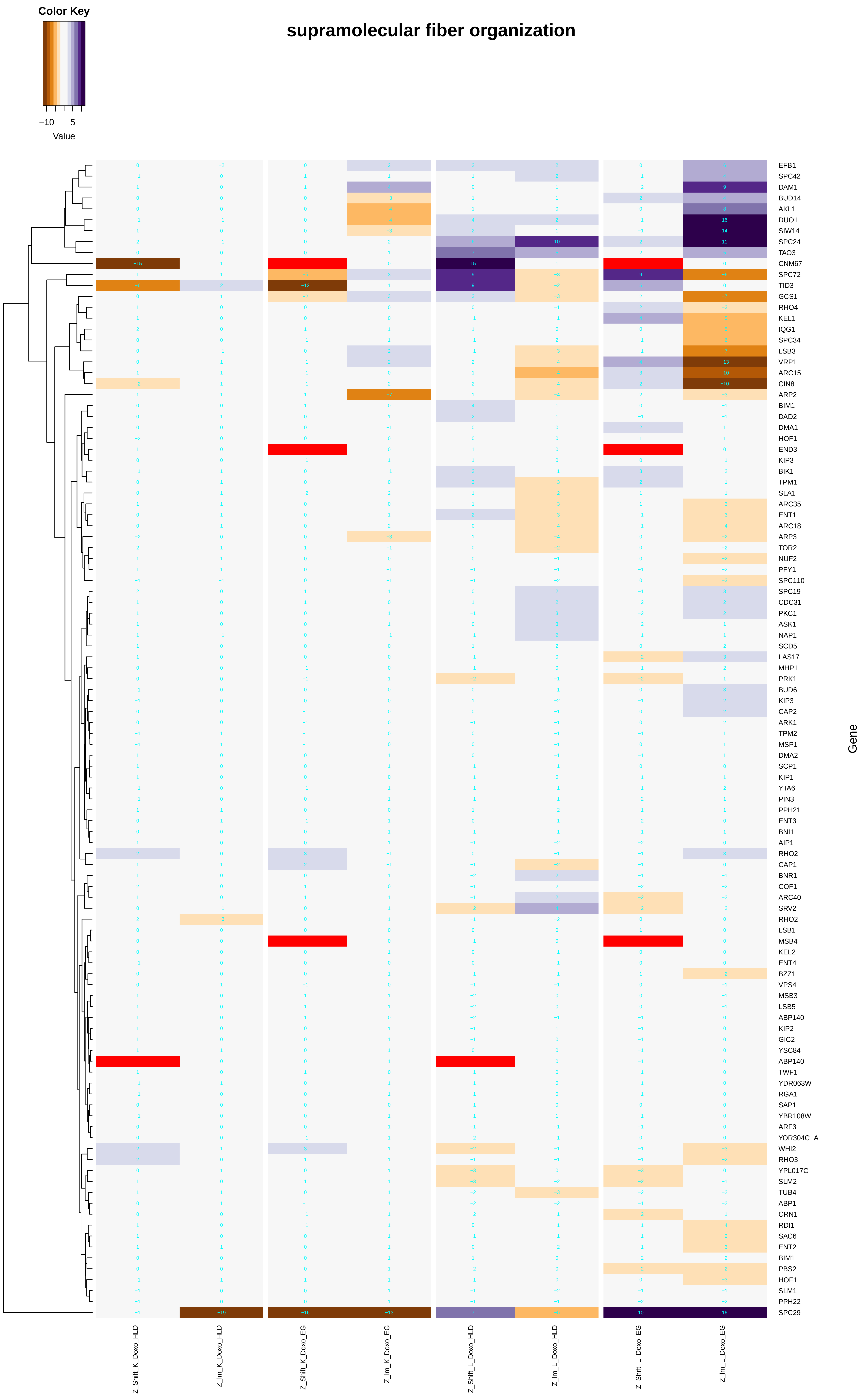
supramolecular fiber organization
Color Key
−10
5
Value
EFB1
SPC42
DAM1
BUD14
AKL1
DUO1
SIW14
SPC24
TAO3
CNM67
SPC72
TID3
GCS1
RHO4
KEL1
IQG1
SPC34
LSB3
VRP1
ARC15
CIN8
ARP2
BIM1
DAD2
DMA1
HOF1
END3
KIP3
BIK1
TPM1
SLA1
ARC35
ENT1
ARC18
ARP3
TOR2
NUF2
PFY1
SPC110
SPC19
CDC31
PKC1
ASK1
NAP1
SCD5
LAS17
MHP1
PRK1
BUD6
KIP3
CAP2
ARK1
TPM2
MSP1
DMA2
SCP1
KIP1
YTA6
PIN3
PPH21
ENT3
BNI1
AIP1
RHO2
CAP1
BNR1
COF1
ARC40
SRV2
RHO2
LSB1
MSB4
KEL2
ENT4
BZZ1
VPS4
MSB3
LSB5
ABP140
KIP2
GIC2
YSC84
ABP140
TWF1
YDR063W
RGA1
SAP1
YBR108W
ARF3
YOR304C−A
WHI2
RHO3
YPL017C
SLM2
TUB4
ABP1
CRN1
RDI1
SAC6
ENT2
BIM1
PBS2
HOF1
SLM1
PPH22
SPC29
Gene
Z_Shift_K_Doxo_HLD
Z_Im_K_Doxo_HLD
Z_Shift_K_Doxo_EG
Z_Im_K_Doxo_EG
Z_Shift_L_Doxo_HLD
Z_Im_L_Doxo_HLD
Z_Shift_L_Doxo_EG
Z_Im_L_Doxo_EG

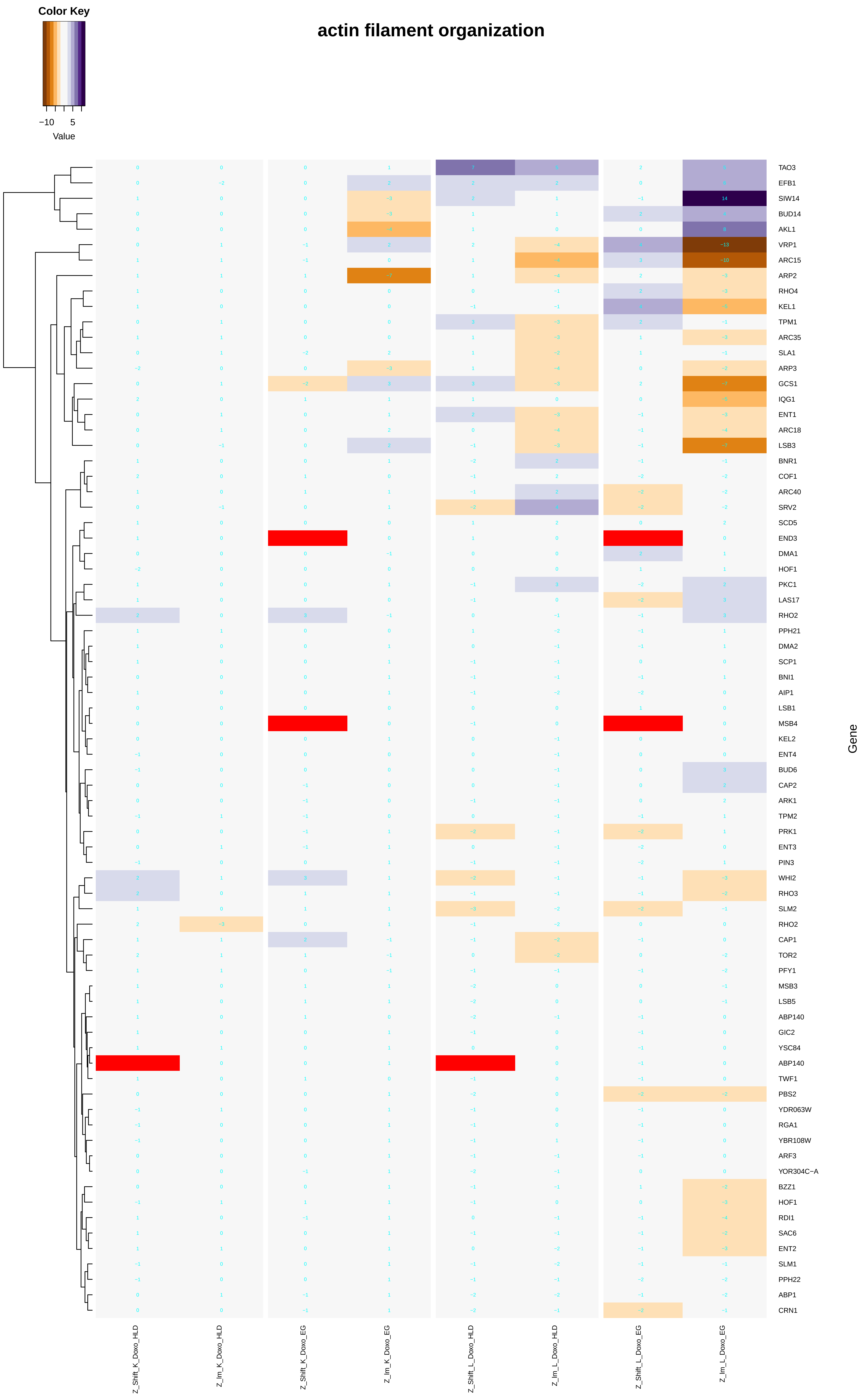
actin filament organization
Color Key
−10
5
Value
TAO3
EFB1
SIW14
BUD14
AKL1
VRP1
ARC15
ARP2
RHO4
KEL1
TPM1
ARC35
SLA1
ARP3
GCS1
IQG1
ENT1
ARC18
LSB3
BNR1
COF1
ARC40
SRV2
SCD5
END3
DMA1
HOF1
PKC1
LAS17
RHO2
PPH21
DMA2
SCP1
BNI1
AIP1
LSB1
MSB4
KEL2
ENT4
BUD6
CAP2
ARK1
TPM2
PRK1
ENT3
PIN3
WHI2
RHO3
SLM2
RHO2
CAP1
TOR2
PFY1
MSB3
LSB5
ABP140
GIC2
YSC84
ABP140
TWF1
PBS2
YDR063W
RGA1
YBR108W
ARF3
YOR304C−A
BZZ1
HOF1
RDI1
SAC6
ENT2
SLM1
PPH22
ABP1
CRN1
Gene
Z_Shift_K_Doxo_HLD
Z_Im_K_Doxo_HLD
Z_Shift_K_Doxo_EG
Z_Im_K_Doxo_EG
Z_Shift_L_Doxo_HLD
Z_Im_L_Doxo_HLD
Z_Shift_L_Doxo_EG
Z_Im_L_Doxo_EG

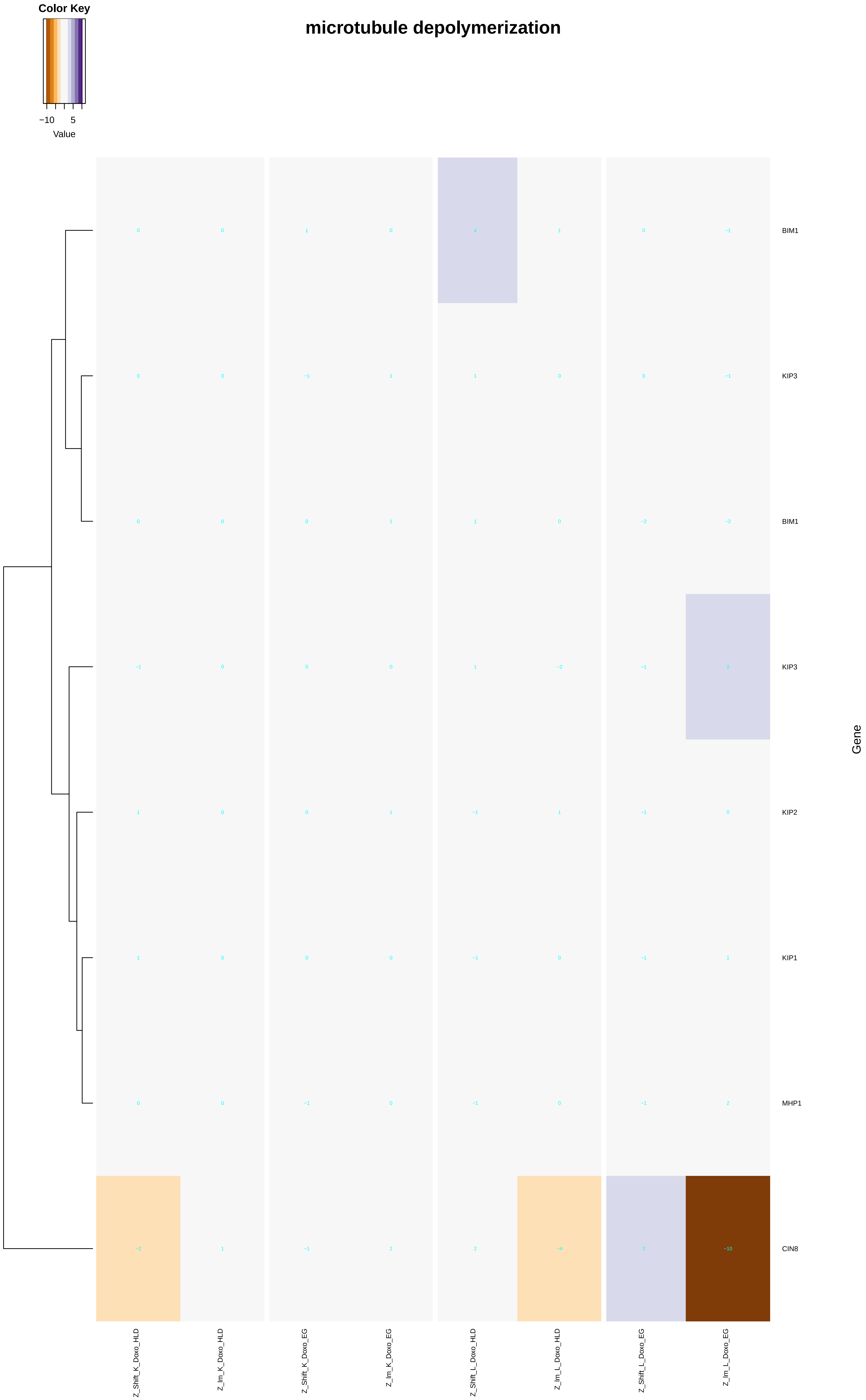
Color Key
−10
5
Value
microtubule depolymerization
BIM1
KIP3
BIM1
KIP3
KIP2
KIP1
MHP1
CIN8
Gene
Z_Shift_K_Doxo_HLD
Z_lm_K_Doxo_HLD
Z_Shift_K_Doxo_EG
Z_lm_K_Doxo_EG
Z_Shift_L_Doxo_HLD
Z_lm_L_Doxo_HLD
Z_Shift_L_Doxo_EG
Z_lm_L_Doxo_EG

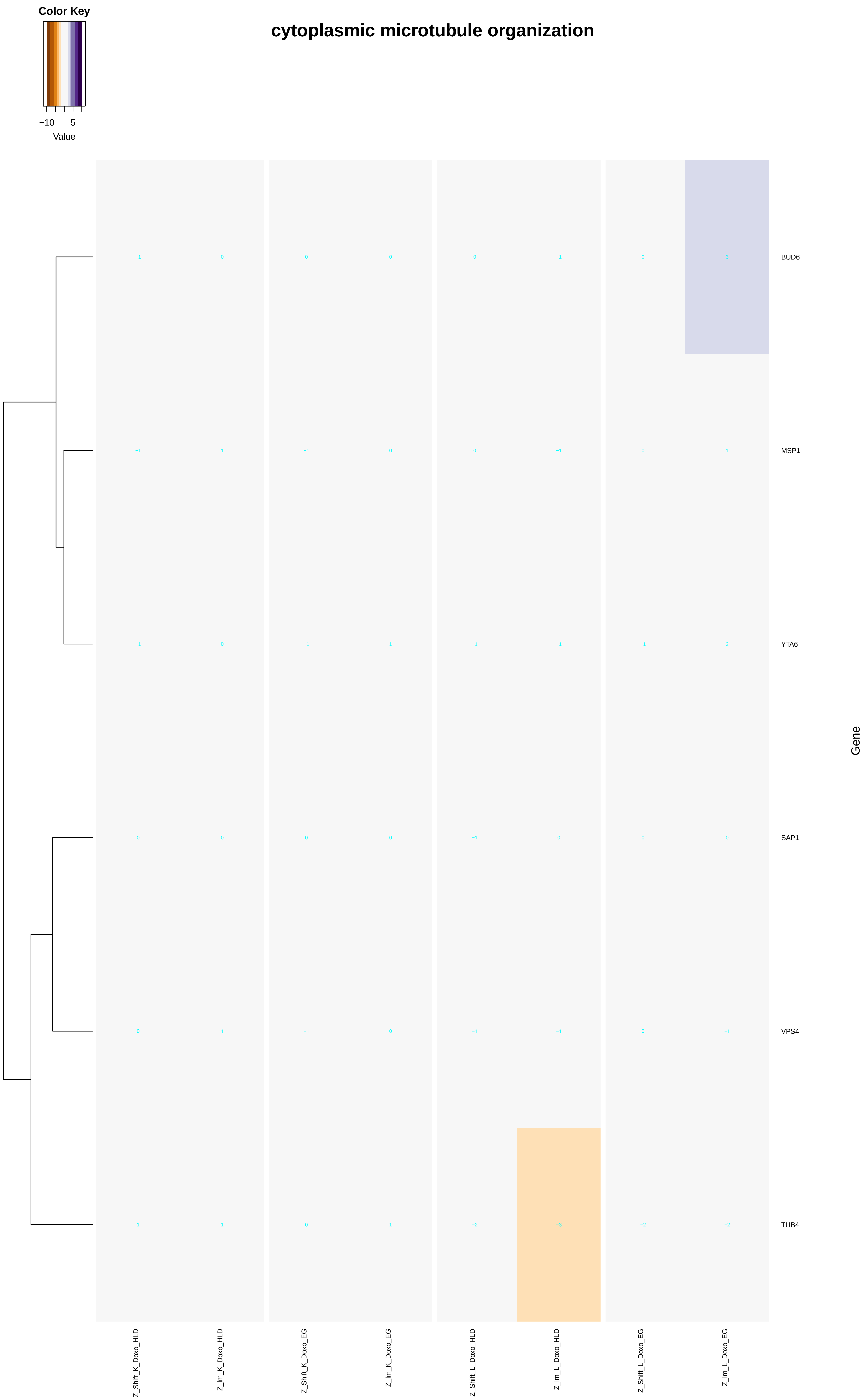
Color Key
−10
5
Value
cytoplasmic microtubule organization
BUD6
MSP1
YTA6
SAP1
VPS4
TUB4
Gene
Z_Shift_K_Doxo_HLD
Z_Im_K_Doxo_HLD
Z_Shift_K_Doxo_EG
Z_Im_K_Doxo_EG
Z_Shift_L_Doxo_HLD
Z_Im_L_Doxo_HLD
Z_Shift_L_Doxo_EG
Z_Im_L_Doxo_EG

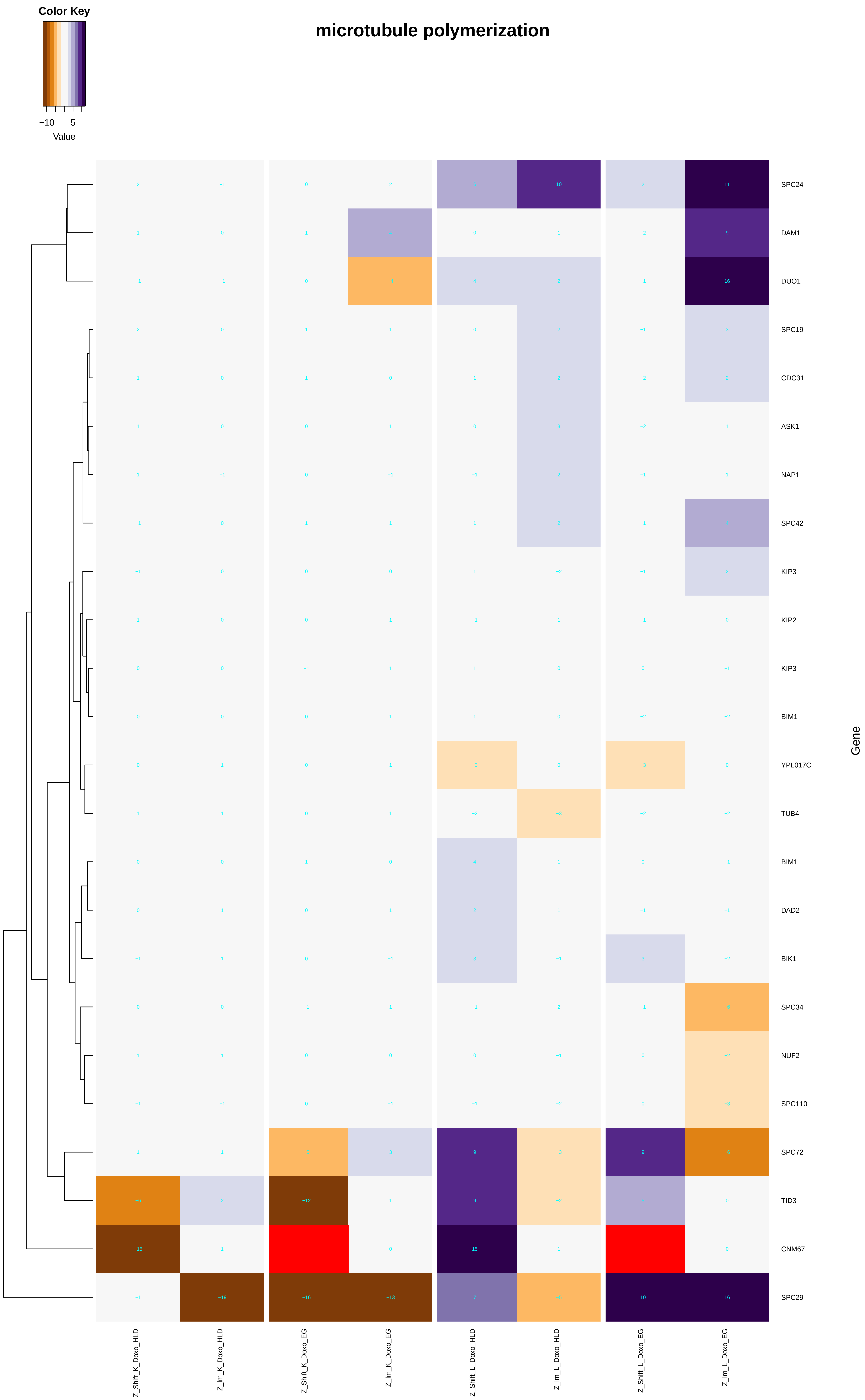
microtubule polymerization
Color Key
−10
5
Value
SPC24
DAM1
DUO1
SPC19
CDC31
ASK1
NAP1
SPC42
KIP3
KIP2
KIP3
BIM1
YPL017C
TUB4
BIM1
DAD2
BIK1
SPC34
NUF2
SPC110
SPC72
TID3
CNM67
SPC29
Gene
Z_Shift_K_Doxo_HLD
Z_Im_K_Doxo_HLD
Z_Shift_K_Doxo_EG
Z_Im_K_Doxo_EG
Z_Shift_L_Doxo_HLD
Z_Im_L_Doxo_HLD
Z_Shift_L_Doxo_EG
Z_Im_L_Doxo_EG

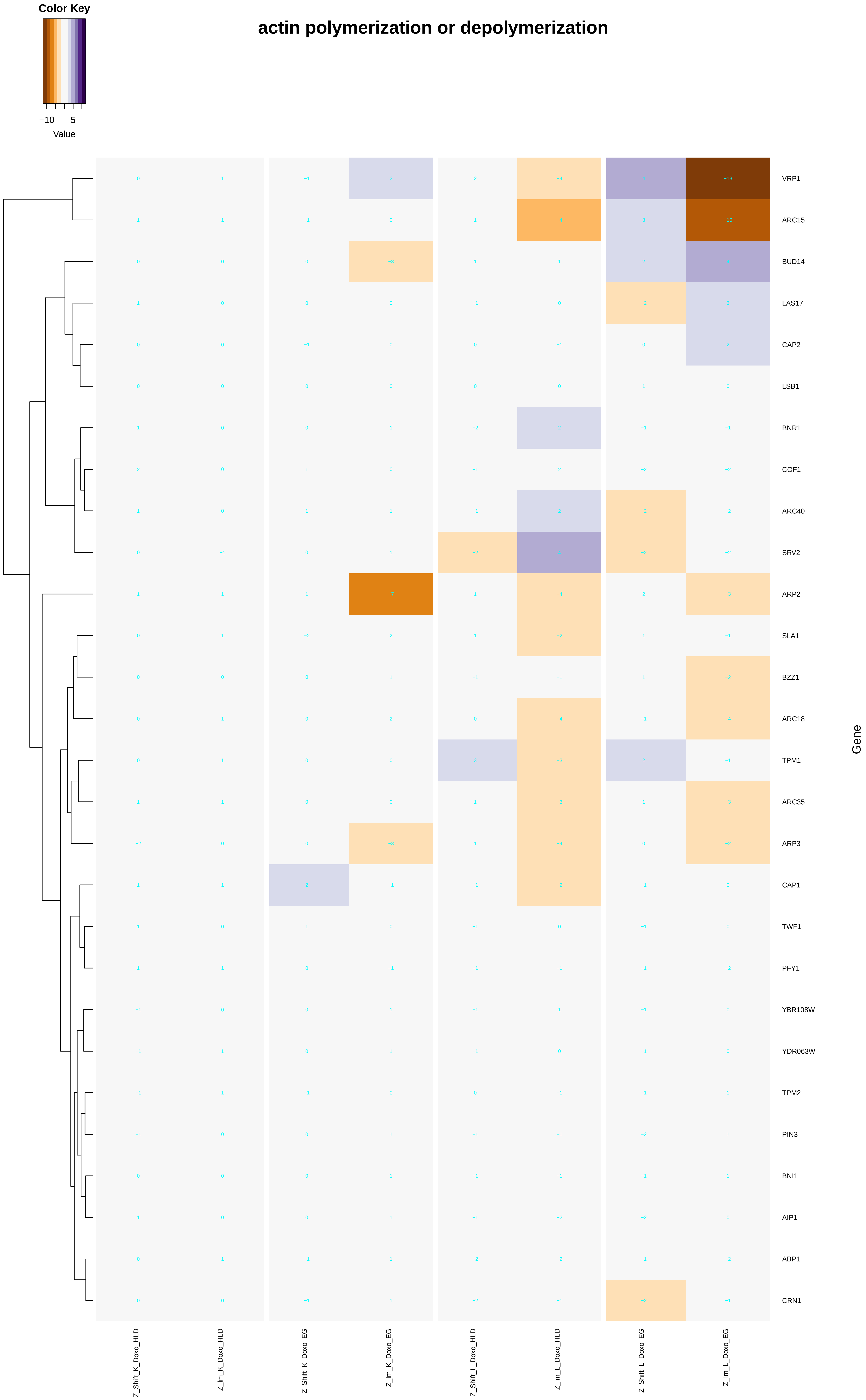
Color Key
−10
5
Value
actin polymerization or depolymerization
VRP1
ARC15
BUD14
LAS17
CAP2
LSB1
BNR1
COF1
ARC40
SRV2
ARP2
SLA1
BZZ1
ARC18
TPM1
ARC35
ARP3
CAP1
TWF1
PFY1
YBR108W
YDR063W
TPM2
PIN3
BNI1
AIP1
ABP1
CRN1
Gene
Z_Shift_K_Doxo_HLD
Z_lm_K_Doxo_HLD
Z_Shift_K_Doxo_EG
Z_lm_K_Doxo_EG
Z_Shift_L_Doxo_HLD
Z_lm_L_Doxo_HLD
Z_Shift_L_Doxo_EG
Z_lm_L_Doxo_EG

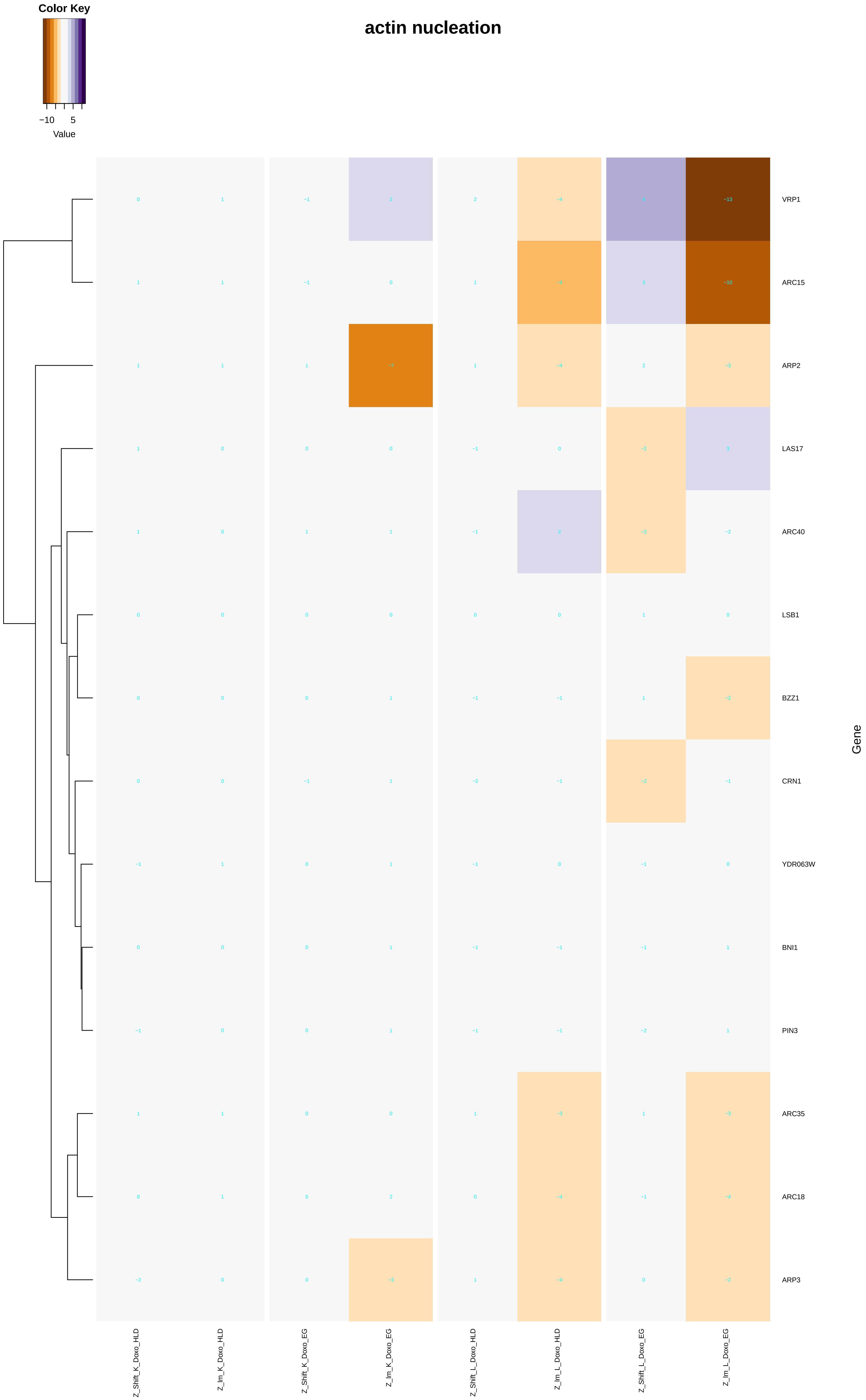

Color Key
−10
5
Value
actin nucleation
VRP1
ARC15
ARP2
LAS17
ARC40
LSB1
BZZ1
CRN1
YDR063W
BNI1
PIN3
ARC35
ARC18
ARP3
Gene
Z_Shift_K_Doxo_HLD
Z_Im_K_Doxo_HLD
Z_Shift_K_Doxo_EG
Z_Im_K_Doxo_EG
Z_Shift_L_Doxo_HLD
Z_Im_L_Doxo_HLD
Z_Shift_L_Doxo_EG
Z_Im_L_Doxo_EG

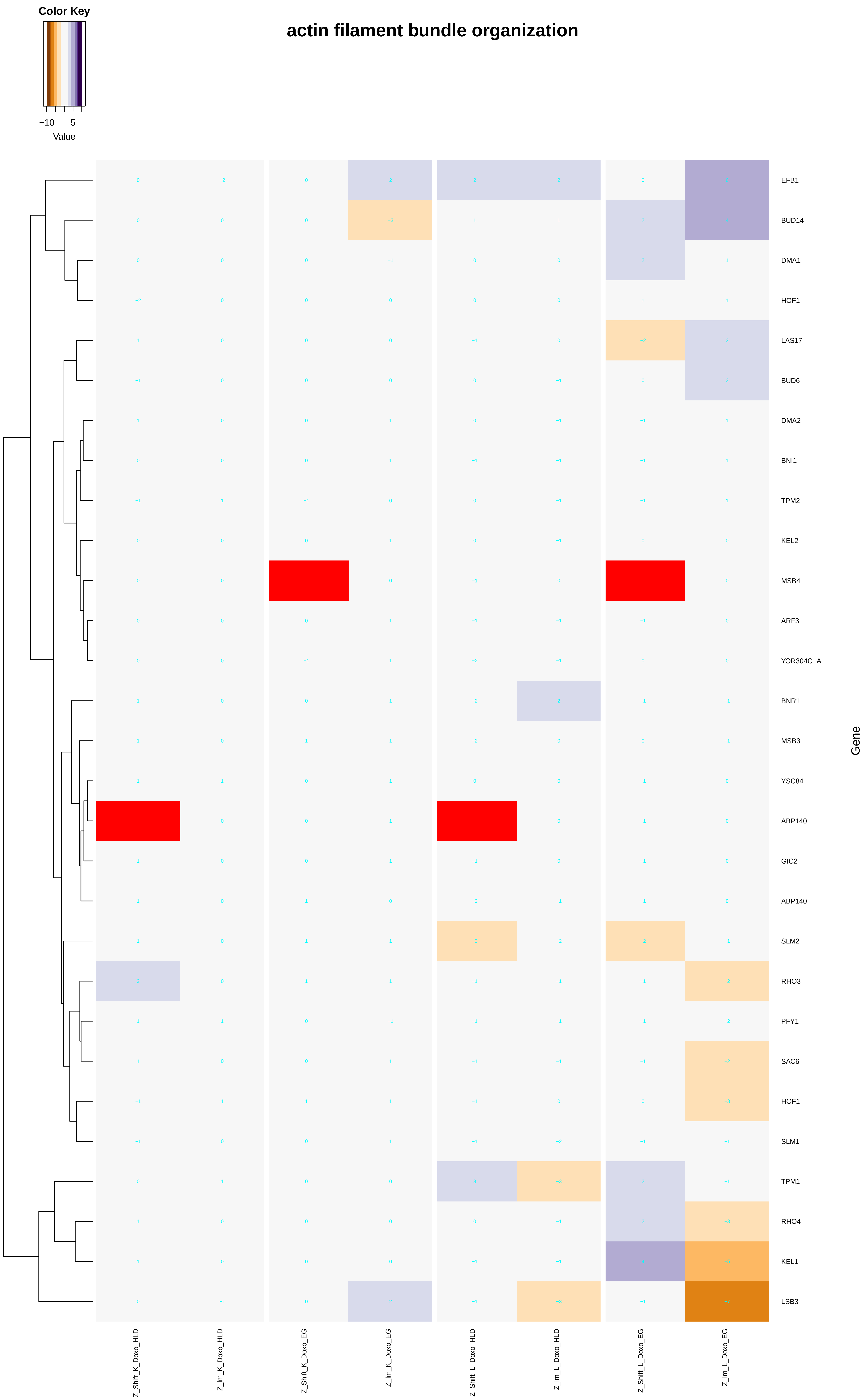
Color Key
−10
5
Value
actin filament bundle organization
EFB1
BUD14
DMA1
HOF1
LAS17
BUD6
DMA2
BNI1
TPM2
KEL2
MSB4
ARF3
YOR304C−A
BNR1
MSB3
YSC84
ABP140
GIC2
ABP140
SLM2
RHO3
PFY1
SAC6
HOF1
SLM1
TPM1
RHO4
KEL1
LSB3
Gene
Z_Shift_K_Doxo_HLD
Z_Im_K_Doxo_HLD
Z_Shift_K_Doxo_EG
Z_Im_K_Doxo_EG
Z_Shift_L_Doxo_HLD
Z_Im_L_Doxo_HLD
Z_Shift_L_Doxo_EG
Z_Im_L_Doxo_EG

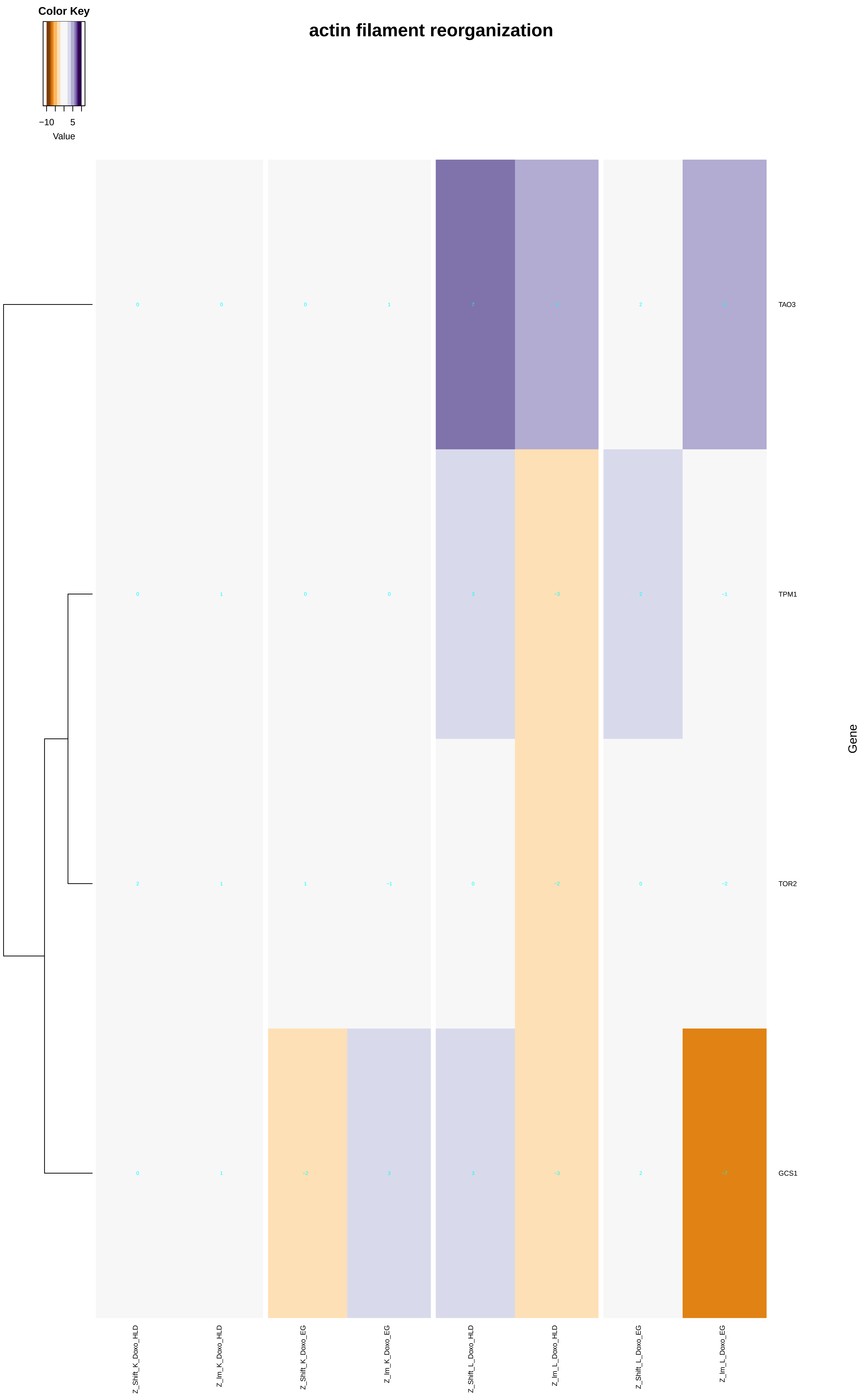
actin filament reorganization
Color Key
−10
5
Value
TAO3
TPM1
TOR2
GCS1
Gene
Z_Shift_K_Doxo_HLD
Z_Im_K_Doxo_HLD
Z_Shift_K_Doxo_EG
Z_Im_K_Doxo_EG
Z_Shift_L_Doxo_HLD
Z_Im_L_Doxo_HLD
Z_Shift_L_Doxo_EG
Z_Im_L_Doxo_EG

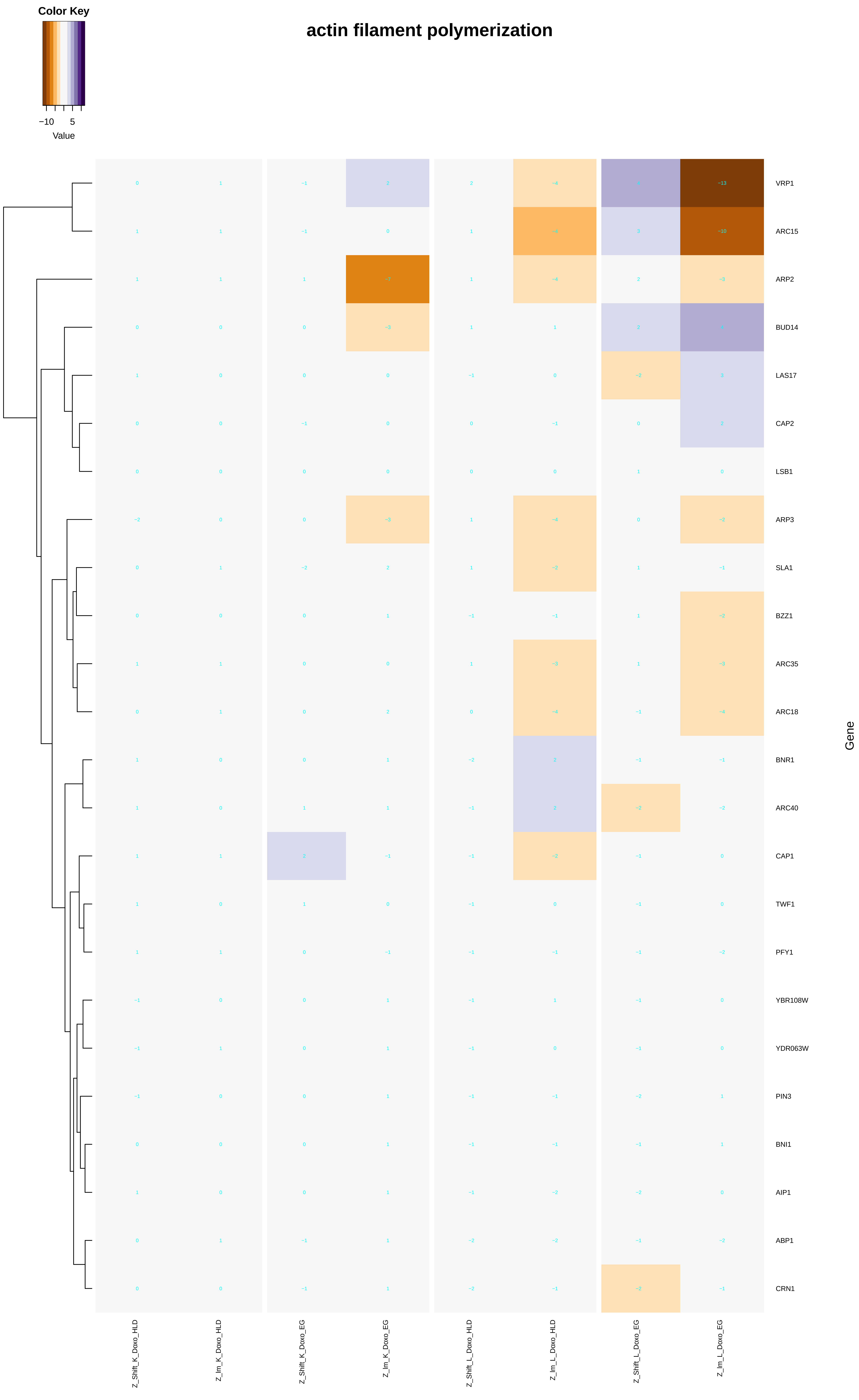
Color Key
−10
5
Value
actin filament polymerization
VRP1
ARC15
ARP2
BUD14
LAS17
CAP2
LSB1
ARP3
SLA1
BZZ1
ARC35
ARC18
BNR1
ARC40
CAP1
TWF1
PFY1
YBR108W
YDR063W
PIN3
BNI1
AIP1
ABP1
CRN1
Gene
Z_Shift_K_Doxo_HLD
Z_Im_K_Doxo_HLD
Z_Shift_K_Doxo_EG
Z_Im_K_Doxo_EG
Z_Shift_L_Doxo_HLD
Z_Im_L_Doxo_HLD
Z_Shift_L_Doxo_EG
Z_Im_L_Doxo_EG

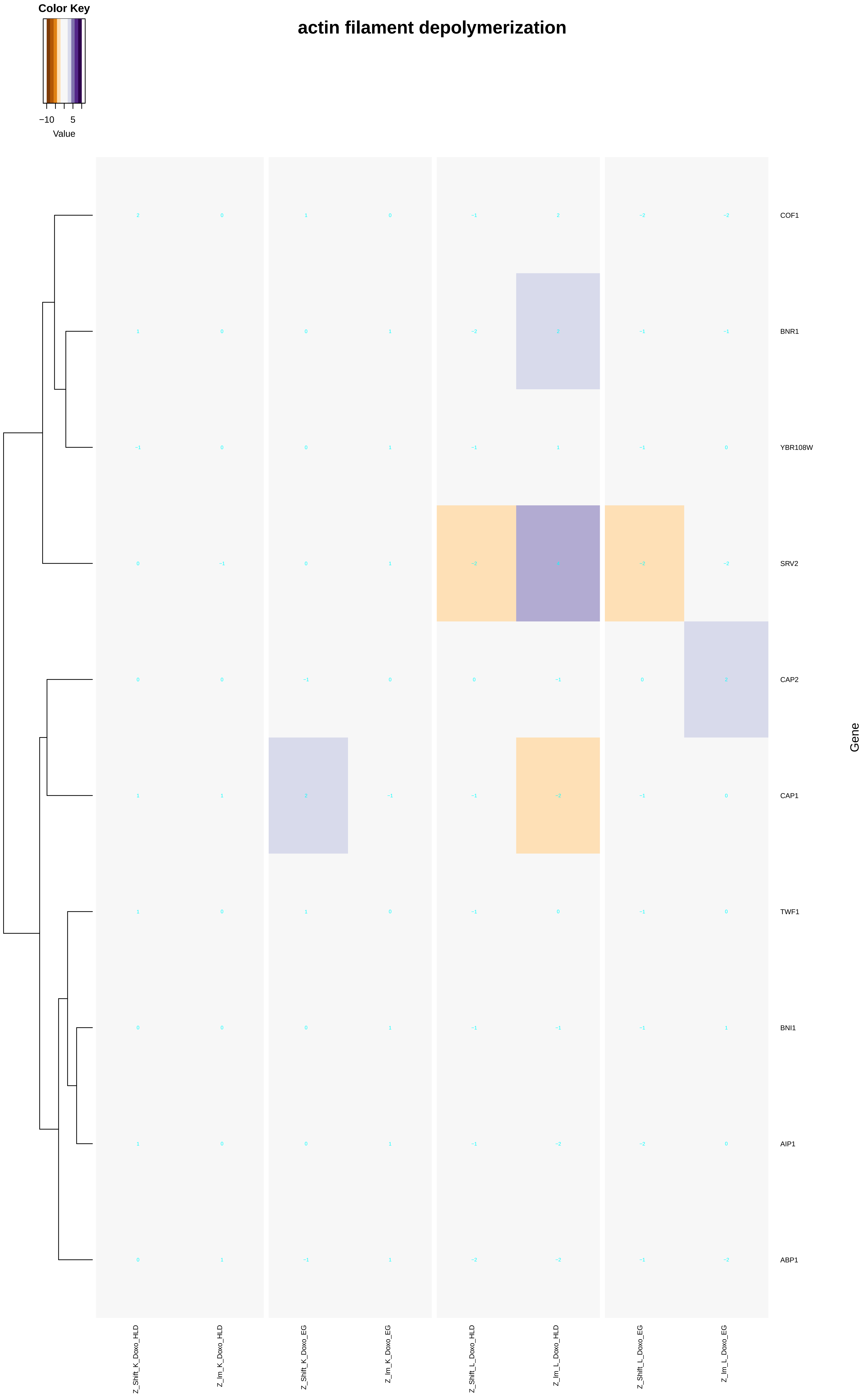
Color Key
−10
5
Value
actin filament depolymerization
COF1
BNR1
YBR108W
SRV2
CAP2
CAP1
TWF1
BNI1
AIP1
ABP1
Gene
Z_Shift_K_Doxo_HLD
Z_lm_K_Doxo_HLD
Z_Shift_K_Doxo_EG
Z_lm_K_Doxo_EG
Z_Shift_L_Doxo_HLD
Z_lm_L_Doxo_HLD
Z_Shift_L_Doxo_EG
Z_lm_L_Doxo_EG

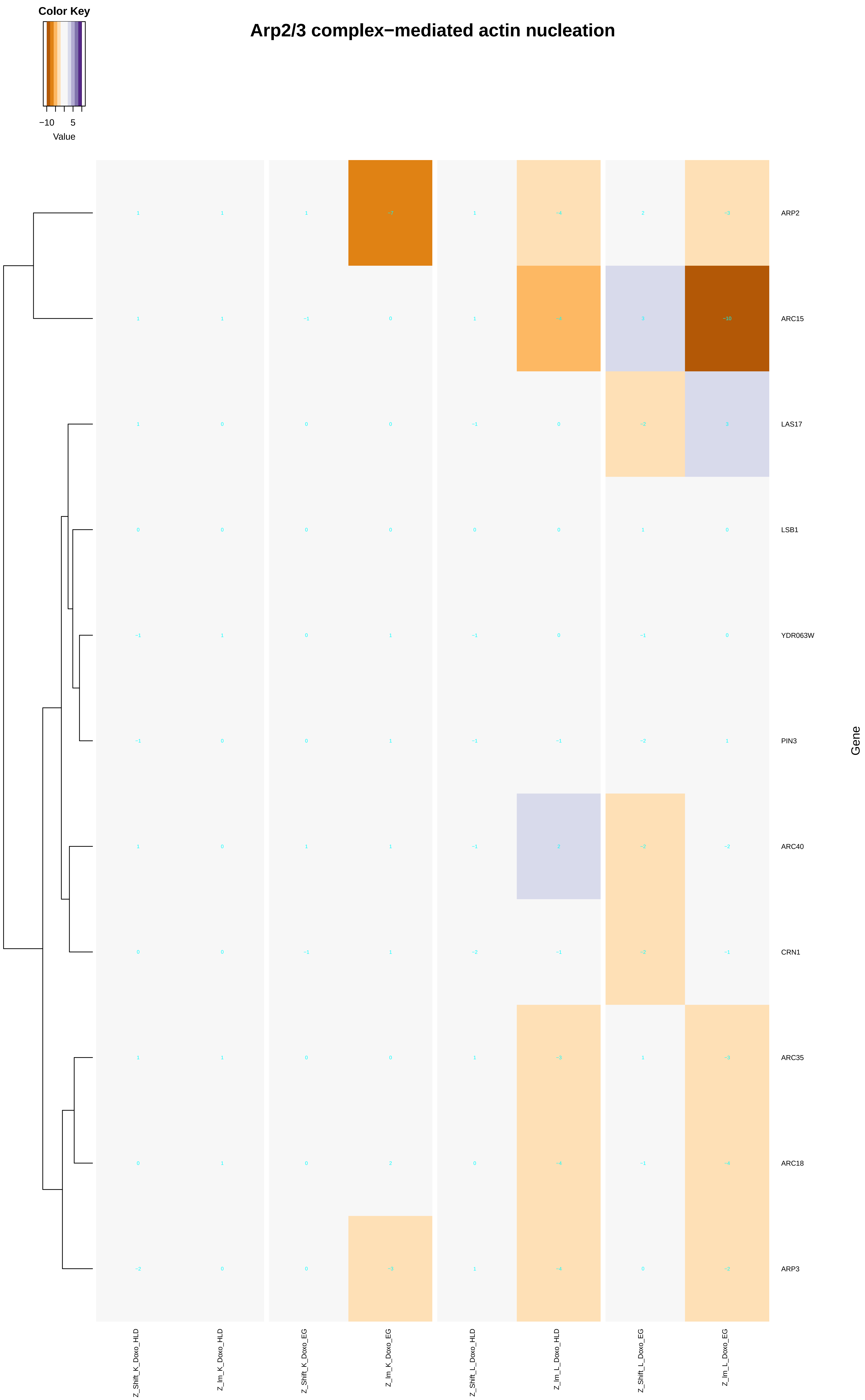
Color Key
−10 5
Value
Arp2/3 complex−mediated actin nucleation
ARP2
ARC15
LAS17
LSB1
YDR063W
PIN3
ARC40
CRN1
ARC35
ARC18
ARP3
Gene
Z_Shift_K_Doxo_HLD
Z_Im_K_Doxo_HLD
Z_Shift_K_Doxo_EG
Z_Im_K_Doxo_EG
Z_Shift_L_Doxo_HLD
Z_Im_L_Doxo_HLD
Z_Shift_L_Doxo_EG
Z_Im_L_Doxo_EG

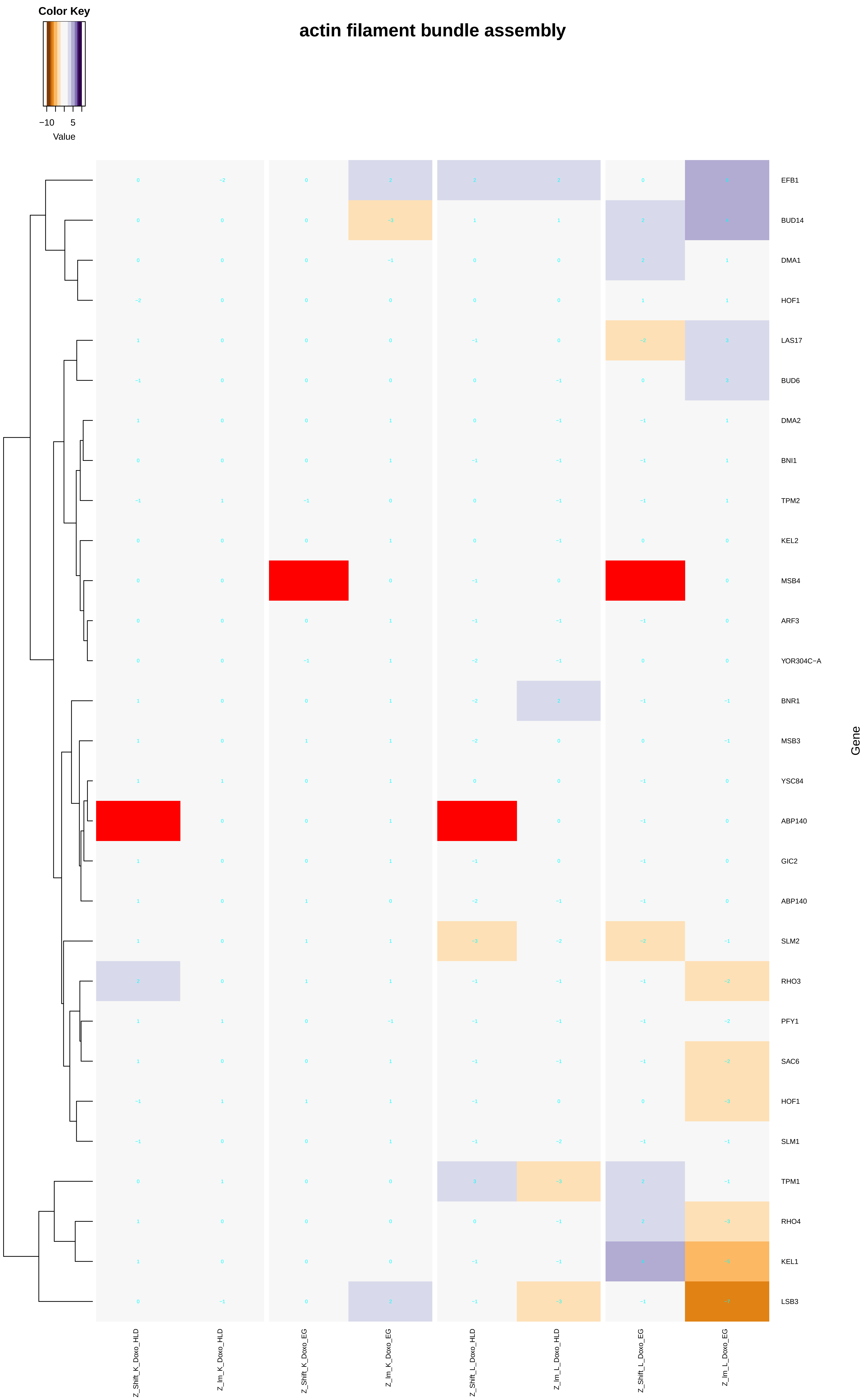
Color Key
−10
5
Value
actin filament bundle assembly
EFB1
BUD14
DMA1
HOF1
LAS17
BUD6
DMA2
BNI1
TPM2
KEL2
MSB4
ARF3
YOR304C−A
BNR1
MSB3
YSC84
ABP140
GIC2
ABP140
SLM2
RHO3
PFY1
SAC6
HOF1
SLM1
TPM1
RHO4
KEL1
LSB3
Gene
Z_Shift_K_Doxo_HLD
Z_Im_K_Doxo_HLD
Z_Shift_K_Doxo_EG
Z_Im_K_Doxo_EG
Z_Shift_L_Doxo_HLD
Z_Im_L_Doxo_HLD
Z_Shift_L_Doxo_EG
Z_Im_L_Doxo_EG

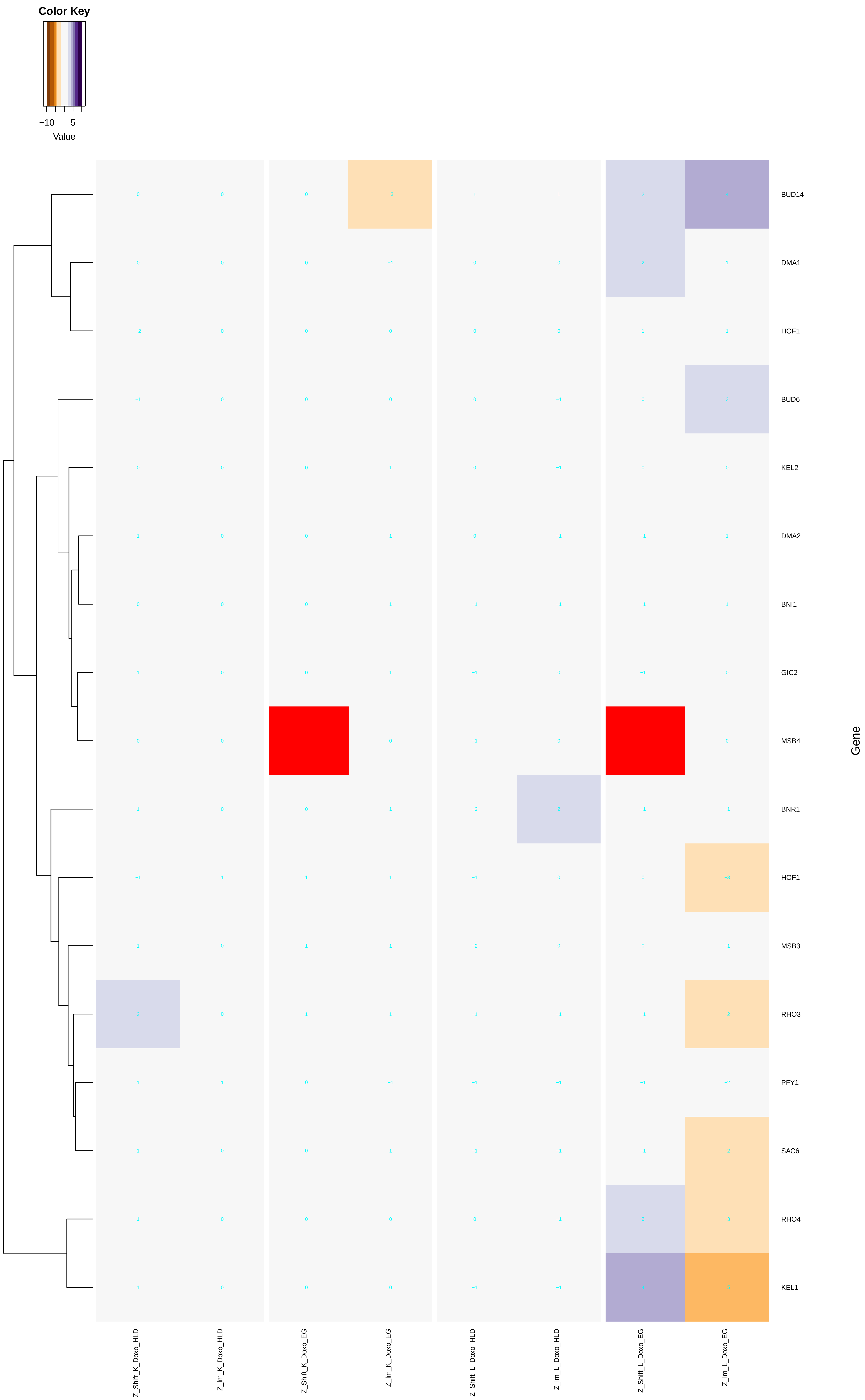

Color Key
−10
5
Value
BUD14
DMA1
HOF1
BUD6
KEL2
DMA2
BNI1
GIC2
MSB4
BNR1
HOF1
MSB3
RHO3
PFY1
SAC6
RHO4
KEL1
Gene
Z_Shift_K_Doxo_HLD
Z_Im_K_Doxo_HLD
Z_Shift_K_Doxo_EG
Z_Im_K_Doxo_EG
Z_Shift_L_Doxo_HLD
Z_Im_L_Doxo_HLD
Z_Shift_L_Doxo_EG
Z_Im_L_Doxo_EG

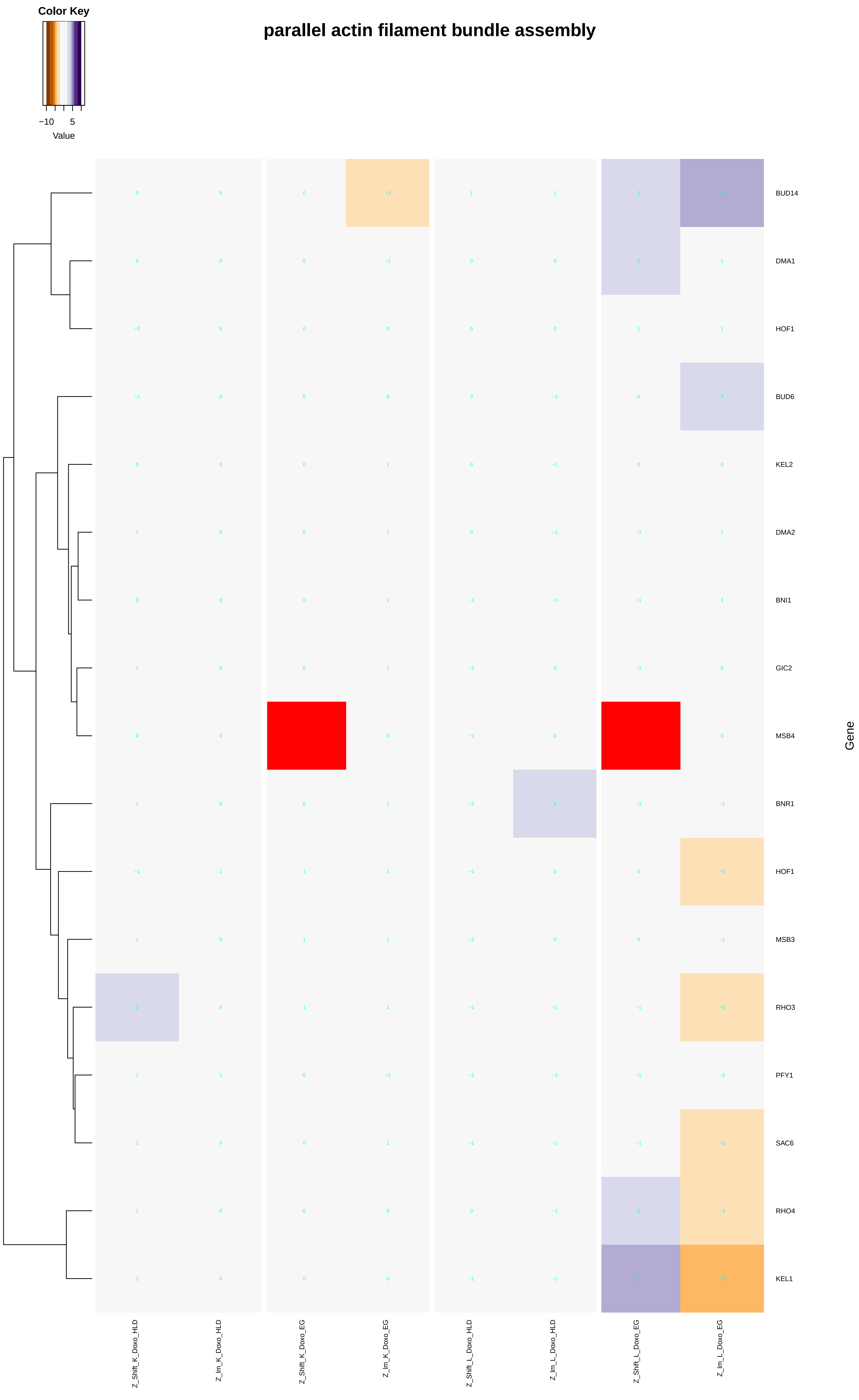

Color Key
−10
5
Value
parallel actin filament bundle assembly
BUD14
DMA1
HOF1
BUD6
KEL2
DMA2
BNI1
GIC2
MSB4
BNR1
HOF1
MSB3
RHO3
PFY1
SAC6
RHO4
KEL1
Gene
Z_Shift_K_Doxo_HLD
Z_Im_K_Doxo_HLD
Z_Shift_K_Doxo_EG
Z_Im_K_Doxo_EG
Z_Shift_L_Doxo_HLD
Z_Im_L_Doxo_HLD
Z_Shift_L_Doxo_EG
Z_Im_L_Doxo_EG

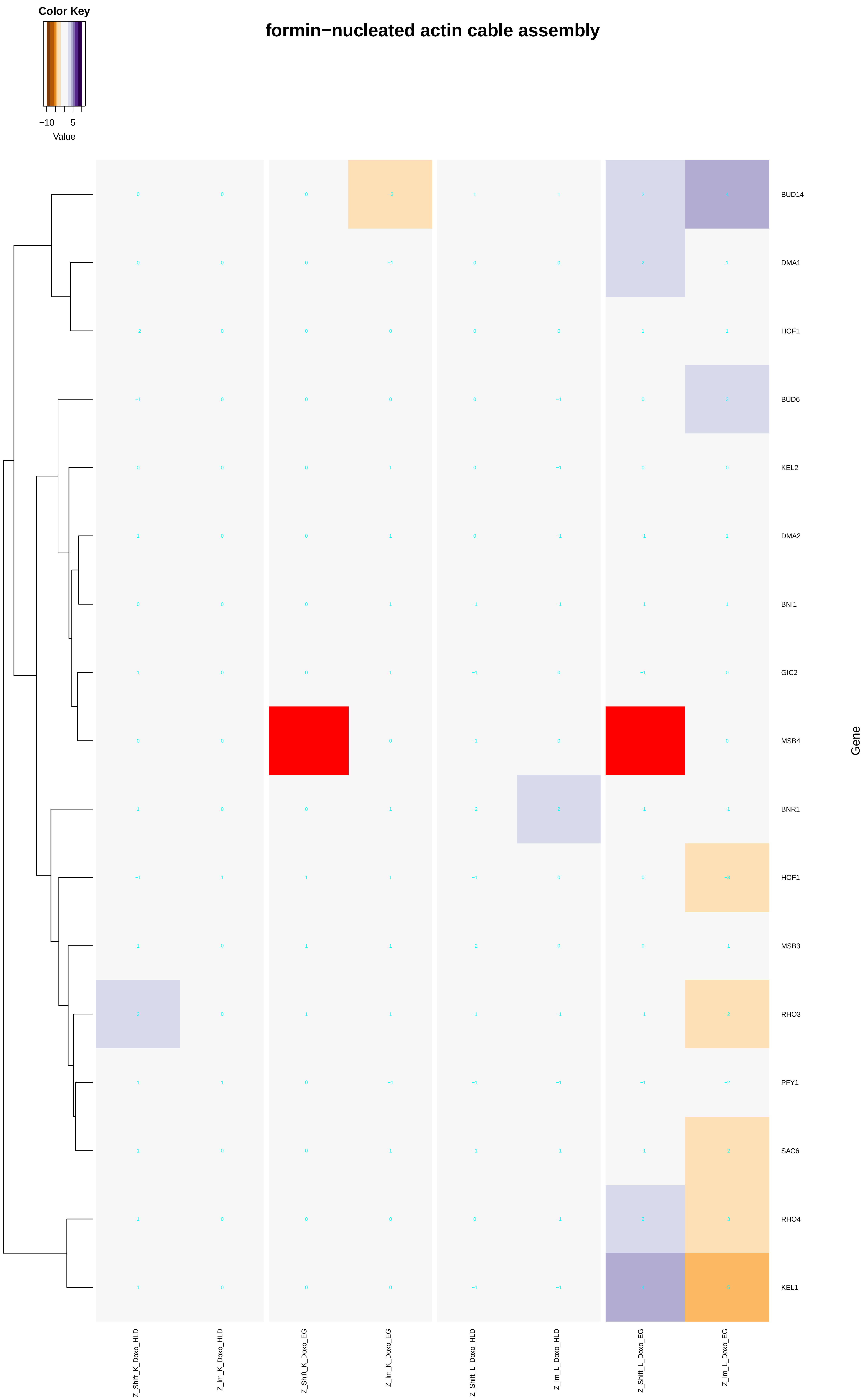
formin−nucleated actin cable assembly
Color Key
−10
5
Value
BUD14
DMA1
HOF1
BUD6
KEL2
DMA2
BNI1
GIC2
MSB4
BNR1
HOF1
MSB3
RHO3
PFY1
SAC6
RHO4
KEL1
Gene
Z_Shift_K_Doxo_HLD
Z_lm_K_Doxo_HLD
Z_Shift_K_Doxo_EG
Z_lm_K_Doxo_EG
Z_Shift_L_Doxo_HLD
Z_lm_L_Doxo_HLD
Z_Shift_L_Doxo_EG
Z_lm_L_Doxo_EG
